# Orthorexia nervosa risk and associated factors among Chilean nutrition students: a pilot study

**DOI:** 10.1186/s40337-022-00529-6

**Published:** 2022-01-11

**Authors:** Manuel Villa, Nicole Opawsky, Sara Manriquez, Nicole Ananías, Pablo Vergara-Barra, Marcell Leonario-Rodriguez

**Affiliations:** 1 Nutrition and Dietetics School, Faculty of Sciences, Universidad Mayor, Temuco, Chile; 2 Departament of Psychiatry and Mental Health, Faculty of Medicine, Universidad de Concepción, Concepción, Chile; 3 Department of Basic Sciences, Center for Molecular Biology and Pharmacogenetics, Faculty of Medicine, Universidad de La Frontera, Temuco, Chile

**Keywords:** Orthorexia nervosa, Nutrition students, Chilean university students

## Abstract

**Background:**

Orthorexia nervosa (ON) is characterized by an excessive, obsessive concern with healthy eating generating psychological complications and even malnutrition at a caloric and protein level. Current evidence suggests that people with greater food knowledge are the most likely to be affected, placing nutrition students as a populational risk group. Since there are no nationwide studies dealing with orthorexia nervosa in this risk group, the present pilot study intends to identify risk factors for orthorexia nervosa in a sample of Nutrition and Dietetics students in Chile.

**Method:**

A descriptive cross-sectional pilot study was done on 90 Nutrition and Dietetics students from a Chilean university, representing 70% of its population. The ORTHO-11-ES instrument was applied to determine ON risk, along with consulting about attitudinal, physical-clinical and social variables. Statistical tests were performed in GraphPad PRISM 8.0®, applying probability ratios and personal correlation, between the sociodemographic variables and the risk of orthorexia nervosa. This study was approved by the university Ethics Committee based on the Helsinki Declaration.

**Results:**

23.3% of the studied population was at risk of suffering ON. Associated variables were being in the second year of their major (OR 2.22), coming from a charter school (OR 3.00) and cohabitation being limited to ≤ 1 person (OR 2.47). Particularly, declared physical activity limits are associated to the risk of suffering ON (Sedentary OR 2.42, Heavy OR 3.53), as well as time spent on the social network Instagram (< 1 h OR 2.77, > 3 h OR 1.80).

**Conclusions:**

There is an ON risk prevalence of 23.3% in the present pilot sample under study, indicating that years of study, cohabitation, secondary educational establishment, physical activity and Instagram use constitute associated factors for the studied condition. Some results vary from international evidence, describing a dual nature in the variables for Instagram time and declared physical activity for ON risk. This study needs replication in more representative samples and longitudinal character with control groups which can confirm the studied elements as ON risk factors.

**Plain English summary:**

Orthorexia nervosa (ON) is an expression created to indicate a possible new eating disorder characterized by excessive and obsessive preoccupation with healthy eating. Some of its most distinctive traits include marked anxiety over food, exaggerated fear over the appearance of some diseases and shame about physical appearance. This ultimately impacts food choice, planning, acquisition, preparation and consumption, creating psychological complications along with some associated with malnutrition. Considering that Nutrition students are an at-risk group, the present pilot study evaluated its prevalence and associated factors in a specific sample in Chile. Conditions associated with the risk of orthorexia nervosa identified in the present study include: number of hours spent using Instagram, limited cohabitation, extreme physical activity, and number of years in the major. These results should be taken cautiously, with their association confirmed in follow-up studies.

## Introduction

Orthorexia nervosa (ON), first described by Steven Bratman in 1997, is defined as a possible new eating disorder characterized by excessive, obsessive worry about healthy eating [1] Although there are no formal diagnostic criteria for Orthorexia Nervosa, several screening tools have been developed to study its clinical characteristics with greater precision. Among its proposed diagnostic criteria are a strong anxiety about affirmative and restrictive dietary practices to aspire to optimal health, accompanied by exaggerated fear over the appearance of diseases and shame about personal physical condition [2]. This mental condition becomes complicated when dietary practices include total restriction of culinary ingredients or even entire food groups, along with fasting, which over time can become increasingly prolonged. It affects food choice, planning, acquisition, preparation and consumption (Table 1), generating both psychological and physical complications, associated with malnutrition at the calorie, protein and micronutrient levels and causing irreversible health damage, as occurs with other eating disorders including Bulimia and Anorexia Nervosa [3, 4].

**Table 1:** Suggested features of Orthorexia Nervosa [36]

Risk population	Teenagers Healthcare professionals Athletes Nutrition Students
Food restriction	Specific culinary ingredients and additives (Sugar, Sodium, Cholesterol) Type of food and preparations (Gluten, GMO, Fried, Fast food) Complete food groups (Carbohydrates and Fats) Time restriction (Fasting)
Attitudes	Dietary supplement use Health security feeling Need for total over-life control Irritability
Complications	Anxiety Body dissatisfaction Depression Malnutrition Other eating disorders

International descriptive studies over the last decade have reported a risk prevalence in the general population of 6.9–90% [5]; however, recent publications project that the prevalence would be between 1 and 3% [6, 7]. Regardless of this, the evidence is still limited to project an increase in the prevalence of risk of this condition. Studies in Chile which have attempted to measure this condition are in early stages and limited to specific age groups. The only report from Jerez et al., is on risk evaluation for ON in secondary students [8], reporting at 30.7% in a sample of 205 adolescents, supporting the premise that this age group is an at-risk population. Similarly, the risk of ON is present in health professionals (58% at risk) [9] and above all, people with greater knowledge about food and nutrition. Studies in Latin America and Western Europe report an ON risk prevalence among Nutrition and Dietetics students of around 70% [10], placing them as the most affected at-risk group, as also occurs with sports science professionals [11].

The previously described points contextualize a worrying situation in Chile, due to the rising numbers of Nutrition students between 2007 and 2015 (65.5%), only exceeded by two other health care majors. 16,000 students are projected for the present year, increasing the size of the at-risk group [12, 13].

Considering how the condition in question can gravely impact the health of its sufferers, and that there are currently no national-level ON studies in this at-risk group, the present pilot study intends to identify the associated risk factors for ON in Nutrition students. We will first apply the ORTHO-11-ES instrument to determine ON risk prevalence, and then characterize sociodemographic, educational and attitudinal aspects. Based on the evaluated variables, we will determine their associations and the ORTHO-11-ES results, in order to establish an important precedent for identifying possible risk factors for ON among nutrition students nationwide.

## Materials and method

A pilot study with a descriptive cross-sectional design was done on Nutrition and Dietetics students at the Universidad Mayor (UM) campus in Temuco, Chile. The sample was determined by Select Statistical Services with 95% reliability and 5% maximum error. Volunteer recruitment was performed institutionally via invitations sent to personal emails, sent out between December 2020 and February 2021. Inclusion criteria were the following: being enrolled in the Nutrition and Dietetics major at UM, knowledge related to personal body composition, and being a regular student in a single level of the major. Exclusion criteria included the presence of any current psychiatric treatment and/or diagnosis, and course load irregularities (among different levels).

Volunteers who agreed to participate in the study signed informed consent online, in order to respond anonymously over the same medium. The data collection instrument asked about the following variables: number of years studied, age, gender, weight, height, secondary school, weekly physical activity frequency, number of cohabitating people and Instagram use. Body mass index was also calculated according to the Quetelet equation to classify nutritional status according to World Health Organization directives [14]. Instagram use was also calculated, derived from data provided by the app settings [15], measured in minutes and then classified in three categories (< 1 h, 1–3 h, and > 3 h). Subsequently, volunteers answered the 11-item Orthorexia Questionnaire Spanish version (ORTO-11-ES instrument), whose objective is to measure ON risk based on 11 self-applied items with a minimum score of 1 and a maximum score of 4. The results achieved were classified as a function of the numerical value of the sum of answers given by study subjects, with a value of 25 or more rated as a negative ON risk, while a value below 25 points characterized the subject as at risk for suffering ON [16].

All data were gathered, organized and processed in Microsoft Excel, and then exported to GraphPad Prism 8.0.0® for Windows (San Diego, California, USA, www.graphpad.com) software for statistical analysis. Distributions, medians and standard deviations were calculated for general background data. Odds ratio and confidence intervals were calculated for discrete quantitative variables and the determination of their ON risk association. Each condition was compared with the remaining sample, providing dichotomous character versus the presence and absence for each of them. For continuous quantitative variables, the confidence interval, Pearson’s r and significance levels were calculated against the ORTO-11-ES score.

This work was undertaken following the guidelines of the Helsinki Declaration and approved by the Ethics Committee at the Universidad Mayor campus in Temuco, Chile.

## Results

90 volunteers who agreed to participate in the study were recruited, amounting to 75% of the total student population in the Nutrition and Dietetics major at Universidad Mayor. This included subjects of both genders, where 12.2% were men and the other 87.8% were women. Regarding participants’ characteristics (Table 2), a similar distribution was obtained for each level, apart from the first year, where only 7 (7.8%) subjects took part, in contrast with the other levels (20–25%). Regarding students’ secondary schooling, only 7.8% of respondents came from public state schools, with the majority of people enrolled in this major coming from charter schools (over 70%). These institutions correspond to educational establishments that receive financial contributions from the government, however their administration is private. It is common for the cost of tuition to be lower than the tuition in a private establishment but higher than the tuition in a public school, which is why this type of schools receive mostly students from the Chilean middle class.

**Table 2:** Sample characterization

	n (%)		n (%)
**Sex**		**Physical activity**	
Male	11 (12.2)	Sedentary	22 (24.4)
Female	79 (87.8)	Light	39 (43.3)
		Moderate	25 (27.8)
**Grade**		Heavy	4 (4.4)
First	7 (7.8)		
Second	23 (25.6)	**Nutritional status**	
Third	20 (22.2)	Underweight	2 (2.2)
Fourth	21 (23.3)	Normal	65 (72.2)
Fifth	19 (21.1)	Overweight	22 (24.4)
**High school origin**		Obesity	1 (1.1)
Public School	7 (7.8)	**Instagram Use**	
Charter School	64 (71.1)	< 1 h	11 (12.2)
Private School	19 (21.1)	1—3 h	57 (63.3)
**Home cohabitation**		3 h or more	22 (24.4)
Alone	2 (2.2)	**ORTO-11-ES results**	
One person	8 (8.9)	Orthorexia Risk	21 (23.3)
Two people	24 (26.7)	Orthorexia Non Risk	69 (76.7)
Three people	26 (28.9)		
Four or more people	30 (33.3)		
***Extracurricular activity***			
Yes	47 (52.2)		
No	43 (47.8)		

A similar situation emerges in cohabitation, where over 80% of study subjects live with at least two people. In contrast, participation in extracurricular activities shows a similar distribution between those who do take part and those who say they do not. For nutritional status, physical activity and Instagram use background, the main responses were normal (72.2%), light activity (43.3%) and 1–3 h of use (63.3%), respectively. Finally, in regards to the main variable measured, 21 out of 90 subjects presented orthorexia nervosa risk, which amounted to 23.3% of the sample.

Regarding the continuous quantitative variables measured (Table 3), both genders presented an average age of 22.2 ± 2.6 years, Body Mass Index (BMI) of 23.2 ± 3 kg/mt^2^, ORTO-11-ES score of 26.8 ± 3.8 and an average daily amount of time on Instagram of 135.5 ± 95.4 min, with a variation coefficient of 70.38%. No statistically significant differences were found regarding differences in these variables by gender among respondents (Table 4).

**Table 3:** Averages of measured variables

	Mean (SD)	CI (95%)	Coefficient of variation (%)
Age (years)	22.2 (2.6)	(21.7–22.8)	11.56
BMI (kg/mt^2^)	23.2 (3.0)	(22.6–23.9)	13.01
Instagram use (min/day)	135.5 (95.4)	(115.5–155.5)	70.38
ORTO-11-ES	26.8 (3.8)	(25.9–27.6)	14.37

**Table 4:** Averages of measured variables by sex

	Men		Women	
	Mean (SD)	CI (95%)	Mean (SD)	CI (95%)
Age (years)	21.73 (2.3)	(20.4–23.1)	22.3 (2.6)	(21.7–22.9)
BMI (kg/mt^2^)	24.15 (2.7)	(22.6–25.7)	23.11 (3.1)	(22.4–23.8)
Instagram use (min/day)	133.7 (40.4)	(109.9–157.6)	135.8 (100.8)	(113.6–158.0)
ORTO-11-ES	25.4 (3.9)	(23.1–27.7)	27 (3.8)	(26.2–27.8)

For the categorized variables and their association with ON risk, their OR and respective confidence intervals were calculated, all as described in Table 5. For the cases of gender, extracurricular activities and nutritional status, no association was found with ON risk or its counterpart. For schooling level, being in the second year of the major showed a probability of ON risk 2.22 times higher than other years, followed by third and fifth year students, but with lower figures. For the socioeconomic level provided by reported secondary education establishment, charter schools were categorically associated with ON risk (OR 3.00, 95% CI: 0.8–11.24) above private schools (OR 0.55, 95% CI: 0.14–2.12). Similarly, cohabitation limited to one person or less (OR 2.47, 95% CI: 0.63–9.76) was a factor by comparison with homes shared with 2, 3 or more people. Other evaluated variables presented a dual nature, since limits on declared physical activity are associated with a risk of suffering ON (Sedentary OR 2.42, 95% CI: 0.84–6.97; Heavy OR 3.53, 95% CI: 0.47–26.72), over light and moderate activity (OR 0.44, 95% CI: 0.15–1.26, and 0.55, 95% CI: 0.18–1.68 respectively). This is also reflected in time spent on Instagram, where lower reported time (OR 2.77, 95% CI: 0.78–9.88) and overuse (OR 1.80, 95% CI: 0.62–5.26), are more associated with ON risk than intermediate use levels (1–3 h).

**Table 5:** Distribution by orthorexia risk

	Orthorexia risk (%)	Orthorexia non risk (%)	OR	CI (95%)
**Sex**				
Male	2 (18.2)	9 (81.8)	0.70	(0.14–3.53)
Female	19 (24.1)	60 (75.9)	1.43	(0.28–7.18)
**Grade**				
First	1 (14.3)	6 (85.7)	0.53	(0.06–4.63)
Second	8 (34.8)	15 (65.2)	2.22	(0.78–6.33)
Third	5 (25.0)	15 (75.0)	1.13	(0.35–3.57)
Fourth	2 (9.5)	19 (90.5)	0.28	(0.06–1.3)
Fifth	5 (26.3)	14 (73.7)	1.23	(0.38–3.93)
**High school origin**				
Public School	0 (0.0)	7 (100.0)	0.00	(0.0–0.0)
Charter School	18 (28.1)	46 (71.9)	3.00	(0.8–11.24)
Private School	3 (15.8)	16 (84.2)	0.55	(0.14–2.12)
**Cohabitation**				
1 person or less	4 (40.0)	6 (60.0)	2.47	(0.63–9.76)
2 people	5 (20.8)	19 (79.2)	0.82	(0.26–2.56)
3 people	7 (26.9)	19 (73.1)	1.32	(0.46–3.76)
4 or more	5 (16.7)	25 (83.3)	0.55	(0.18–1.68)
**Extracurricular activity**				
Yes	11 (23.4)	36 (76.6)	1.01	(0.38–2.68)
No	10 (23.3)	33 (76.7)	0.99	(0.37–2.64)
**Physical activity**				
Sedentary	8 (36.4)	14 (63.6)	2.42	(0.84–6.97)
Light	6 (15.4)	33 (84.6)	0.44	(0.15–1.26)
Moderate	5 (20.0)	25 (100.0)	0.55	(0.18–1.68)
Heavy	2 (50.0)	2 (50.0)	3.53	(0.47–26.72)
**Nutritional status**				
Underweight	0 (0.0)	2 (100.0)	0.00	(0.00–0.00)
Normal	15 (23.1)	50 (76.9)	0.95	(0.32–2.81)
Overweight	6 (27.3)	16 (72.7)	1.33	(0.44–3.98)
Obesity	0 (0.0)	1 (100.0)	0.00	(0.00–0.00)
**Instagram use (per day)**				
< 1 h	5 (41.7)	7 (58.3)	2.77	(0.78–9.88)
1–2 h	5 (18.5)	22 (81.5)	0.67	(0.22–2.06)
2–3 h	4 (13.8)	25 (86.2)	0.41	(0.13–1.37)
3 h or more	7 (31.8)	15 (68.2)	1.80	(0.62–5.26)

**Fig. 1:**
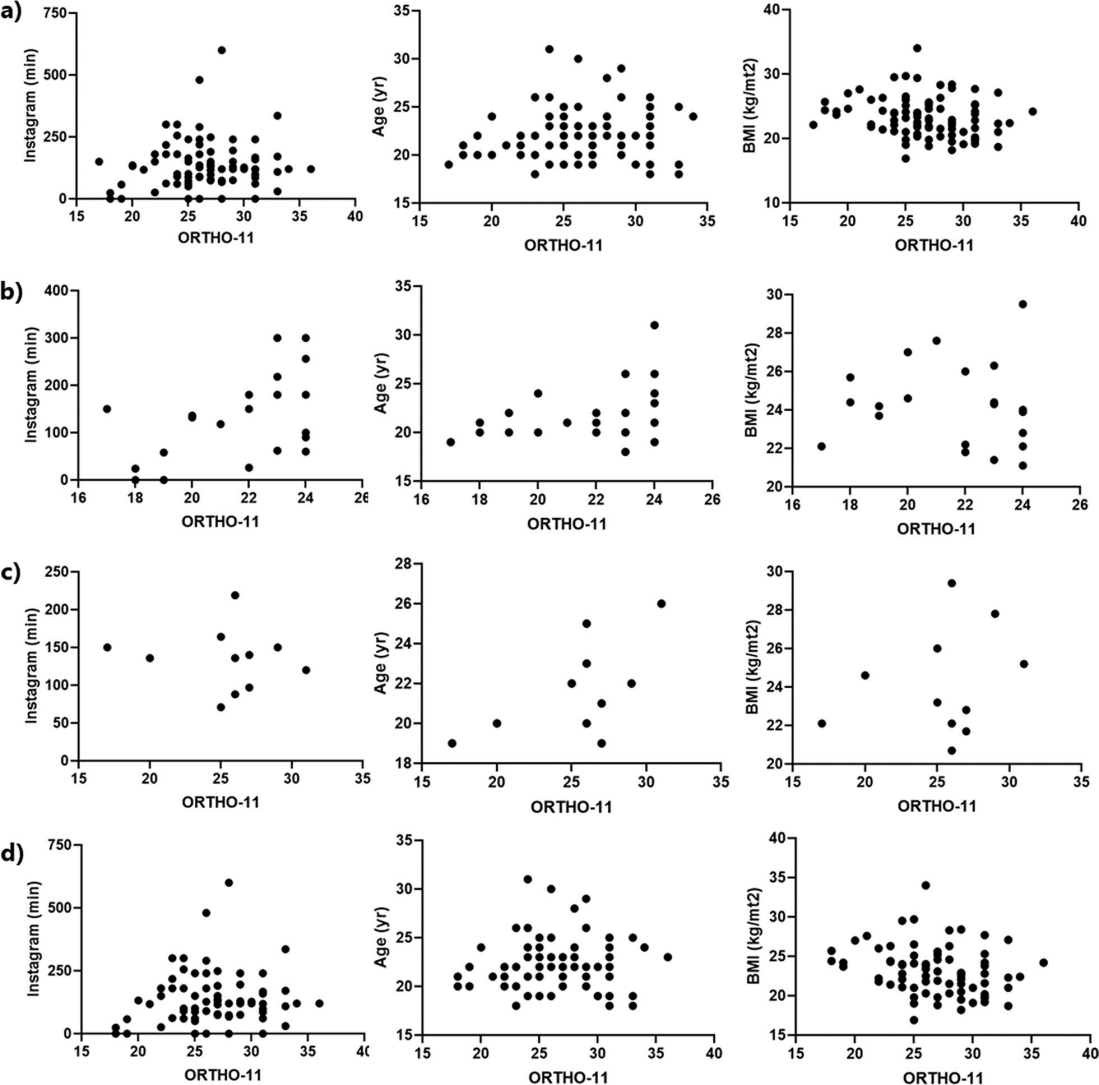
Correlation between ORTHO-11 results, Age, BMI and Instagram use

Finally, regarding the correlations determined for continuous quantitative variables (Fig. 1), a positive correlation was reported (r = 0.49), which was statistically significant (*p* = 0.022) between the ORTO-11-ES score of people with ON risk and daily minutes spent on Instagram. A negative correlation was detected for the BMI variable among women (r = −0.27 and *p* = 0.014) and in the general sample (r = −0.23 and *p* = 0.029). In men, age correlated positively with ORTHO-11-ES score, but was not statistically significant (Table 6).

**Table 6:** Correlation between ORTHO 11 results and quantitative continuous variables

Instagram				Age				BMI			
All	< 25	Men	Women	All	< 25	Men	Women	All	< 25	Men	Women
0.06	0.49*	−0.11	0.07	0.07	0.39	0.59	−0.004	−0.23*	−0.08	0.26	−0.27*

^*^p < 0.05

## Discussion

Being a nutrition student could be an important risk factor for developing orthorexia nervosa according to different international studies [10, 17–20]. Considering the rise in enrollment rates and increase in the number of students in Chile, it is necessary to develop incentives which allow for identifying associated factors which promote the condition and allow us to establish lines of action for prevention and management. In this line, the present study constitutes an important precedent as the first work of this nature at the national level, and along with two studies done in Brazil in previous years, the only work undertaken in South America.

The main results include the evidence of an ON risk prevalence rate of 23.3%, greater than described for the general population or the rate described by Escobar et al. [16] among Peruvian medical students. Other rates were reported at between 30 and 80% among nutrition students, however, all studies used different versions of the ORTHO instrument, making comparisons unviable. A publication by Dunn et al. declared that these values were overestimated, apart from indistinct use of the terminology related with diagnosis and risk, demonstrating the need for greater caution when comparing these results. It would be interesting to analyze which out of all the ORTHO versions generated has a better diagnostic capacity, especially because of the ORTHO-15 results which report rates above 50% of evaluated samples. It is also important to mention that these evaluations mainly focused on European populations, where sociodemographic and sanitary realities are very different from the reality of Chilean university students [21–23]. The ORTHO-11-ES used in the present study corresponds to a validated version in the Spanish-speaking population and at the South American level. Confirmatory factor analysis supported 11 elements and 3 domains as the best fit model, using the comparative fit index and Tucker-Lewis index metrics. In addition, it was validated by the in health students from a Peruvian population [16].

One major goal of the present study was to identify factors associated to ON risk in nutrition students. Particularly, being in the second year is more associated with ON risk than other levels. Even as the major progresses, and nutritional knowledge therefore expands, the risk diminishes. The present result could be mediated by the courses done during the second year of the major, which involves a sudden inclusion of disciplinary courses associated with self-measurement of body segments, including Nutritional Status Evaluation, along with quantitative food evaluation in areas including Basic Nutrition and Food Planning [24, 25]. The decrease in ON risk prevalence per level as the years advance (Table 5) could be due to the orientation of the following courses: the characterization of food and ideal body composition. These focus more on the qualitative value of food and healthy criteria contributing to a non-reductionist training. Understanding that the qualitative value of food refers to the sensations that food evokes after perceiving its flavors and smells, it is necessary to incorporate these types of variables to standardize the study subjects when researching in this area. It would be interesting to compare this with other studies; however, there is no risk characterization per level in the current literature, making our results unprecedented in this sense.

Regarding the other associated factors, in prior studies secondary educational establishment type was identified as a variable associated with ON risk. During 2015, Jerez et al. [8], evaluated a cohort of secondary school students who came from different schools, reporting higher rates in public state schools. This reoccurred in our study, where students from private schools had only a 15% risk of ON, which was lower than charter school students at 28% (OR 3.00). Considering this, along with Chilean inequality levels, it would be interesting to evaluate in future studies whether food and nutrition education is a protective factor against ON risk during adolescence and adulthood, as it has been reported with other educational interventions against Anorexia and Bulimia [26, 27]. It could also be possible that, depending on socioeconomic level, home diet quality will show concrete differences with knowledge acquired in higher education, placing individuals in undesired vulnerable conditions which are hard to remedy [28].

Another factor associated with ON risk observed in this sample is limited cohabitation with one person or less, which places perceived social support as a variable to consider, as reported by various publications discussing eating disorders more generally. In addition, living with more people would allow the exchange of experiences through food [29].

However, when queried about extracurricular activities, a lack of engagement with them did not constitute a factor associated with ON risk. This could be influenced by the variability of activities performed, with an understanding that not all are associated with increasing social support, including practicing individual sports and exercise [30]. Simultaneously, the current strict social distancing measures in Chile due to the pandemic could be a limiting factor for positive aspects of extracurricular activities considering how in-person activities have been changed for remote events [31]. Furthermore, nutritional status defined by body mass index, was not an element for consideration, since the odds ratio is similar in all students, regardless of their body mass index ranking. Which could be related to specific social pressure in this population group, with their general preoccupation for body composition and nutritional status, regardless of what their classification may be.

The results for the variables of physical activity and use of Instagram are interesting, since the associations determined by the odds ratio were concentrated in the extreme classifications of each category (in sedentary / heavy population and less than one hour / more than 3 h). This situation presents the fact that both sedentary status and heavy physical activity are factors on which to focus. The same thing happens with social network use, where spending less than 1 h per day and spending 3 or more hours per day were both factors associated with ON risk on this sample. These data suggest that avoidance as a coping strategy for this problem among sedentary subjects who present limited interaction with social media; the latter point is unprecedented in comparison with international evidence [15]. On the other extreme, subjects who undertook excessive physical activity and overused Instagram could be influence by beauty standards created in gyms and social networks, including “Fitspiration” dynamics [32].

An association was found between different variables and the risk of orthorexia nervosa according to the OR values, however, it is important to analyze these data with caution, considering the width of the confidence intervals calculated. It will be important in a future occasion to include a greater number of nutrition students from other universities, to define the real magnitude of the association between the variables studied in the present work. Regarding the correlations between the continuous quantitative variables, it was possible to show that the number of minutes used in Instagram correlates positively with the ORTO-11-ES score only when the population at ON risk is considered (Fig. 1). This situation could be explained by the avoidance that the population with a higher ON risk spending less time using the social network that causes dissatisfaction with their diet. These results disagree with international evidence [15], and it is interesting to discuss how social networks affect the continent to which the evaluated subjects belong. When evaluating according to age, no correlation with the ORTO-11-ES score is observed. Age was a variable little explored in the present topic. Previous reports have only evaluated orthorexia in the young adult population [33], and currently no studies have been published that consider older adults, so it is not possible determine the impact of this variable. Interestingly, at the BMI level, negative correlations were found, unlike when this variable was worked ordinally and the OR values were determined. Correlations assume that the higher the BMI, the lower the ORTO-11-ES and risk of ON scores are obtained. These results support what was described by Novara et al. [34], but it will depend on whether the sample studied is clinical or not.

It should be mentioned that there are important limitations on this study considering the number of subjects evaluated. However, the sample is a representative number for the quantity of people enrolled in an average university Nutrition and Dietetics major in Chile.

Also, it is necessary to detail that ON does not constitute a diagnosis as itself, and that it corresponds to an evolving concept. Along these lines, it is necessary to improve the psychometric properties of the instruments that aim to measure the risk of ON. The latter being an important limitation of the present study, since the instrument has not yet been validated in Chile.

Therefore, the present pilot initiative presents several interesting variables worth investigating which had not been identified in other studies, such as major year and duality in social media use. However, considering the nature of the study, interpretation should be taken with caution considering the design used.

Future studies should try to have more representativeness, expanding the sample size and consider schools from different regions around the country to reproduce the geographical and populational heterogeneity of Chile [35].

Furthermore, a longitudinal design contrasted with a control population would allow us to evaluate the risk or protective factor condition of the variables which were studied, vis-à-vis ON risk in this specific population.

## Conclusion

The present pilot study reported that physical activity, study years, cohabitation, secondary school type and Instagram use are factors associates with the condition of ON. This study needs to be replicated in samples which better represent Chilean national diversity, and longitudinal studies need to be done with control groups which can confirm these elements as factors for Orthorexia Nervosa risk, which in the future may be able to establish guidelines for prevention strategies.

## Data Availability

The data sets used and analyzed during this study are available from the corresponding author upon personal request.

## Abbreviations

ON: Orthorexia nervosa
BMI: Body mass index
